# Addiction in the time of COVID-19: Longitudinal course of substance use, psychological distress, and loneliness among a transnational Tyrolean sample with substance use disorders

**DOI:** 10.3389/fpsyt.2022.918465

**Published:** 2022-07-25

**Authors:** Kilian Lommer, Timo Schurr, Beatrice Frajo-Apor, Barbara Plattner, Anna Chernova, Andreas Conca, Martin Fronthaler, Christian Haring, Bernhard Holzner, Christian Macina, Josef Marksteiner, Carl Miller, Silvia Pardeller, Verena Perwanger, Roger Pycha, Martin Schmidt, Barbara Sperner-Unterweger, Franziska Tutzer, Alex Hofer

**Affiliations:** ^1^Division of Psychiatry I, Department of Psychiatry, Psychotherapy, Psychosomatics and Medical Psychology, Medical University Innsbruck, Innsbruck, Austria; ^2^Department of Psychiatry, General Hospital of Bolzano, Sanitary Agency of South Tyrol, Bolzano, Italy; ^3^Therapy Center Bad Bachgart, Sanitary Agency of South Tyrol, Rodengo, Italy; ^4^Department of Psychiatry and Psychotherapy B, State Hospital Hall in Tyrol, Hall in Tyrol, Austria; ^5^Department of Psychiatry, General Hospital of Brunico, Sanitary Agency of South Tyrol, Brunico, Italy; ^6^Department of Psychiatry and Psychotherapy A, State Hospital Hall in Tyrol, Hall in Tyrol, Austria; ^7^Department of Psychiatry, County Hospital Kufstein, Kufstein, Austria; ^8^Department of Psychiatry, General Hospital of Merano, Sanitary Agency of South Tyrol, Merano, Italy; ^9^Department of Psychiatry, General Hospital of Bressanone, Sanitary Agency of South Tyrol, Bressanone, Italy; ^10^Department of Psychiatry, County Hospital Lienz, Lienz, Austria; ^11^Division of Psychiatry II, Department of Psychiatry, Psychotherapy, Psychosomatics and Medical Psychology, Medical University Innsbruck, Innsbruck, Austria

**Keywords:** COVID-19, substance use disorders, psychological distress, loneliness, social support

## Abstract

**Introduction:**

Next to an increased use of alcohol, the current pandemic has been associated with increased psychological distress among the general population. Research on its effects on individuals suffering from substance use disorders (SUD) is scarce. This study aimed at expanding the existing literature on this topic with a focus on the impact of loneliness and perceived social support.

**Methods:**

Sixty-eight people diagnosed with SUD according to ICD-10 from the Austrian state of Tyrol and from the Italian Province of South Tyrol who had been treated in a psychiatric hospital in 2019 and one hundred and thirty-six matched reference subjects of the same regional background participated in an online survey. Sociodemographic variables and scores on the Brief Symptom Checklist, the Three-Item Loneliness Scale, and the Multidimensional Scale of Perceived Social Support were collected at baseline and 5 months thereafter. Baseline took place after the first wave, while follow-up largely coincided with the second wave of the pandemic.

**Results:**

Among both patients and the matched reference group, substance use as a means to feel better facing the pandemic rose and predicted higher levels of psychological distress. Patients were less likely to receive specific care at follow-up than at baseline and presented with a significantly higher prevalence of clinically relevant psychological distress and loneliness than the matched reference group at both assessment times. Among both groups, psychological burden remained unchanged over time. Perceived social support was generally significantly higher in the matched reference group than in patients. Loneliness and, to a lesser degree, low perceived social support predicted psychological distress.

**Conclusion:**

These findings emphasize the need of preventive and educational measures regarding substance use behavior for both individuals suffering from SUD and those without mental health disorders.

## Introduction

The COVID-19 pandemic has affected societies and individuals throughout the world as it spread globally in early 2020, leading to substantial numbers of infection and death. The first cases in Italy and Austria were confirmed on January 31st and February 8th, 2020, respectively. Several weeks later, first fatalities occurred and case numbers rose substantially, prompting the responsible authorities to implement public health measures, including the disruption of various aspects of public and social life. The ensuing psychosocial stress has had severe repercussions on the general population's wellbeing (1, 2). Notably, among the general population, various measures of mental health and psychological distress have deteriorated in the course of the pandemic (3–6), which held true for healthcare professionals involved in the pandemic response as well (7, 8). Individuals suffering from psychiatric diseases have been considered particularly vulnerable in that regard (9–11).

The individual response and the capability to cope with a stressor can differ substantially between individuals. Substance consumption as a strategy to alter one's emotional state when facing stressors (12–14) has been linked to a problematic substance use at later points (14, 15) and to disadvantageous consequences for mental health (16–18). While some research has provided a more ambiguous picture (8, 19, 20), a number of investigations have shown that in the course of the pandemic, the use of alcohol (21–25) and other addictive substances (24, 26) has increased among the general population across different countries. This has in turn been associated with poor mental health (22–25). Being subjected to lockdown measures has been linked to an increased risk of hazardous drinking patterns and of alcohol use disorder (27). Furthermore, substance use appeared connected to an increased risk and severity of COVID-19 infections (28, 29) and an increase in alcohol-related emergencies has been reported (29).

Social support has generally been identified as a protective variable in the face of different kinds of stressful events (30–33) such as the recent global health crisis (34, 35). Conversely, loneliness has been shown to be a relevant predictor of distress and psychopathology (36–40). In the context of the current pandemic, the available literature suggests an association of loneliness and increased distress (41–45) as well as an association of loneliness and increased alcohol consumption (25, 46). For example, one of our recent studies in a Tyrolean sample of the general population (1) found female sex, low income, and being single as well as the use of alcohol or other substances to be associated with high psychological distress and loneliness.

Based on third variables (e.g., neuroticism, traumatic live events) (47), alcohol use disorder appears connected to a higher propensity for psychological distress (47–51). At the same time, it has been associated with low resilience (52, 53) and reduced stress tolerance (54), suggesting a particular vulnerability of affected individuals facing the pandemic. However, research on the repercussions of the current crisis on individuals suffering from substance use disorder (SUD) is relatively scarce. Communications by professionals as well as qualitative research has pointed to challenges in the treatment of SUD, particularly in regard to increased psychosocial stress and reduced access to treatment services (55–59). While some research has found an ambiguous development of consumption patterns in individuals with alcohol use disorder (AUD) (60–62) and SUD (63), a number of publications related an increased risk of relapse in patients with AUD (64–67) and an increase of addictive behavior in other SUD (68, 69). This has been linked to increased psychosocial strain due to COVID-related stressors (62, 64, 67–69) with loneliness in particular having been identified as a factor associated with higher craving (68). To expand these findings, we undertook a longitudinal study on psychological distress and its relationship with loneliness and social support in individuals suffering from SUD in comparison to a community reference group without self-reported mental health disorders. We hypothesized that substance use would increase across participant groups and that increased substance use would be associated with increased psychological distress. Furthermore, we hypothesized that patients would be more severely affected in terms of psychological distress than matched references, that the attendance of mental health infrastructure would decrease over time, and that measures of social integration would be valid predictors of wellbeing during the pandemic.

## Materials and methods

### Sample

We undertook a longitudinal online survey in people with and without a history of mental health disorders in the regions of Tyrol (Austria) and South Tyrol (Italy). South Tyrol has been annexed by Italy after World War I; however, the population has similar characteristics and is comparable with Tyrol in many ways (socioeconomic context, healthcare system, etc.) (70).

Individuals with a mental health disorder aged 18 and above who had been treated in one of a number of psychiatric wards in Tyrol and South Tyrol in 2019 were invited by letter to complete the online survey. Altogether, 1,285 patients diagnosed with SUD were invited to participate, of which 87 enrolled. 68 individuals completed both baseline and follow-up surveys and were included in the analyses of the current report. 46 (67.6%) had a primary diagnosis of SUD according to ICD-10, and 22 (32.4%) had a secondary diagnosis of SUD. A majority (*n* = 53, 77.9%) of the patient sample had been diagnosed with alcohol use disorder, with smaller numbers of participants diagnosed with (in descending frequency) disorders due to the use of sedatives and hypnotics, multiple substances, opioids, cannabinoids and tobacco. The primary diagnosis of the majority of those with a secondary diagnosis of SUD was a mood disorder. Diagnoses were confirmed using chart information.

Additionally, a matched reference group from the general population was recruited through advertising in social and print media, flyers, and e-mail lists. Due to the public nature of parts of the recruitment scheme, it was not possible to determine the number of eligible individuals that were reached but decided not to take part in the investigation. A total of 1,646 people participated in the baseline survey [results obtained in the Tyrolean subsample are reported in Tutzer et al. (1) and Chernova et al. (71)] and were asked to provide an email address to be reminded for follow-up assessment. Participants, who met the following inclusion criteria were selected for further processing: 18 years or older, no self-reported previous diagnosis of a mental health disorder (including SUD) as well as currently no psychopharmacological and/or psychotherapeutic treatment. The obtained sample (*N* = 1,197), included 481 individuals which completed all relevant questionnaires (i.e., sociodemographics, BSCL, TILS, and MSPSS) at baseline and follow-up.

Since SUD prevalences are strongly associated with age and sex (72, 73), we selected a matched reference sample based on the patients' age and sex distribution. Therefore, consistently five age groups (≤34 years; 35–43 years; 44–50 years; 51–58 years; ≥59 years) among female and male participants from the previously obtained reference sample were generated. The selection procedure was realized by randomly picking (fair coin toss) individuals, falling into the corresponding age and sex group, until the intended ratio was reached.

Due to the age and sex distribution within both samples as well as an intended increase in power regarding further statistical analyses, the ratio of the patient group and matched reference group was chosen to be 1:2 (74–76). Consequently, this resulted in a total sample of 68 patients diagnosed with SUD and 136 individuals meeting the aforementioned inclusion criteria.

The study was approved by the Ethics Committee of the Medical University Innsbruck, Austria (Approval Number 1147/2020) and by the Ethics Committee of the Sanitary Agency of South Tyrol, Italy (Approval Number 83-2020). All participants provided informed consent online. At the end of the survey, they received a downloadable information sheet on professional support numbers and addresses.

### Procedures

Data was gathered at two time points. In Tyrol, the baseline survey (T1) was conducted between June 26th, 2020 and September 13th, 2020, in South Tyrol between September 07th, 2020 and November 22nd, 2020. The 5-month follow-up (T2) took place between November 30th, 2020 and January 24th, 2021 (Tyrol) and between February 8th, 2021 and April 4th, 2021 (South Tyrol), respectively. The different time periods for the two regions were owed to organizational reasons, however, the time interval between surveys was equal in both countries.

Data acquisition was conducted *via* the Computer-based Health Evaluation System (CHES) (77). In the following, only data relevant to the present report will be presented. Further data provided by study participants (including individuals suffering from other mental health disorders) have been (1, 11, 71) or will be presented in other reports.

Firstly, sociodemographic data was recorded, including age, sex, educational level, relationship and employment status, and household income. In addition, participants were asked whether they had used alcohol or other substances since the outbreak of the pandemic in order to feel better. Clinical data was recorded for patients, including psychiatric diagnoses, time passed since initial diagnosis and first inpatient treatment, and currently attended modalities of psychiatric care.

Psychological distress was assessed using the Brief Symptom Checklist (BSCL) (78), a 53-item questionnaire with a Likert scale design and items to be scored from 0 (not at all/no distress) to 4 (extremely/very strong distress). Nine dimensions of psychological distress are measured, including anger-hostility, anxiety, depression, paranoid ideation, phobic anxiety, psychoticism, somatization, interpersonal sensitivity, and obsessive-compulsiveness. From the single items, an aggregate score referred to as Global Severity Index (GSI) is derived. In accordance with the authors' recommendations, an age- and sex-based normative T-Score of ≥63 was used as cut-off to consider values indicative of clinically relevant psychological distress.

Loneliness was measured *via* the Three-Item Loneliness Scale (TILS), a brief scale with favorable psychometric properties (79). It consists of the questions “How often do you feel that you lack companionship?,” “How often do you feel left out?,” and “How often do you feel isolated from others?” to which the answers “Often,” “Some of the time,” and “Hardly ever or never” are suggested. Thus, scores between 3 and 9 can be attained. In accordance with previous research, scores of 5 or 6 were considered indicative of moderate loneliness, whereas scores ≥7 were defined as indicating severe loneliness (38).

Lastly, we assessed perceived social support using the Multidimensional Scale of Perceived Social Support (MSPSS) (80). It is a 12-item scale evaluating social support on the three sub-dimensions of family, friends, and significant others. Items were scored on a Likert scale with scores ranging from 1 (“strongly disagree”) to 5 (“strongly agree”). Scores >50% indicate high perceived support.

### Statistical analysis

IBM SPSS 27 (81) was used for statistical analyses. Sociodemographic data, patient and COVID-19-related aspects, critical BSCL *t*-values, and means of outcome variables within the patient and the matched reference group at baseline were compared by non-parametric test procedures for dichotomous and categorical/non-normally distributed metric variables (Fisher's exact test; Mann-Whitney-U test). Parametric tests were used for metric variables (t-test), when normally distributed. McNemar tests were conducted for baseline and follow-up comparison within the respective group. Initially, a pre-analysis was conducted aiming to find possible associations between the GSI of the BSCL measured at baseline and at follow-up and perceived social support (MSPSS) as well as loneliness (TILS) at baseline by means of Spearman correlations. Furthermore, these variables were analyzed regarding substance use during the COVID-19 pandemic by means of non-parametric Mann-Whitney-U test. Correlation coefficients can be interpreted as follows: *r* < 0.10 no correlation; *r* = 0.10–0.29 low correlation; *r* = 0.30–0.49 moderate correlation, and *r* ≥ 0.50 high correlation (82). For the primary analyses complete-case repeated measures ANCOVA were employed in order to account for interactions and main effects of covariates and multiple assessments on psychological distress. The patient and matched reference group were used as factor, time as variable of multiple assessments (baseline and follow-up), and substance use, perceived social support (MSPSS), and loneliness (TILS) as covariates. Reported GSI score mean values were adjusted for included covariates within the repeated measures ANCOVA model. Furthermore, a second model controlling for the matching variables (age and sex) was analyzed, in order to account for possibly introduced associations between the matching factor and the outcome. Effect sizes expressed by η^2^ can be interpreted as: η^2^ ≥ 0.01 small effect; η^2^ ≥ 0.06 medium effect, and η^2^ ≥ 0.14 large effect. Since a complete-case analysis might produce increased standard errors and significance levels compared to the larger sample, first-order autoregressive (AR1) linear mixed models were employed to validate the results obtained by variance analyses. This method uses maximum-likelihood estimation to handle missing data. Additionally, due to distinct measurement periods in Tyrol and South Tyrol at baseline and follow-up, these analyses were used to account for both the COVID-19 seven-day incidence rate and the time of measurement. Therefore, in addition to the variables described in the repeated measures ANCOVA, these two variables were included. After transforming the date of participation into the respective month, it was used as a factor. The COVID-19 7-day incidence rate at the respective day of participation in the corresponding region was included as a covariate.

### Power calculations

Power analysis was conducted with G^*^Power (version 3.1.9.2) (83). It is based on the assumption of type 1 error (alpha = 0.05) and power of 1 – beta = 0.8. Calculated f effect sizes were transformed into η^2^. The sample of 68 patients diagnosed with SUD and 136 individuals from the matched reference group is sufficiently large to detect in a correlation analysis effect sizes of *r* ≥ 0.33 and *r* ≥ 0.23, respectively. Concerning repeated measures ANCOVA between factors analysis, with a total sample of 188 participants, an effect size of η^2^ ≥ 0.021 can be detected. For within factors analyses, effect sizes of η^2^ ≥ 0.038 (patients), and η^2^ ≥ 0.014 (reference group) are detectable. Regarding within and between interaction analyses, the sample is sufficiently large to detect an effect size of η^2^ ≥ 0.010.

## Results

### Baseline characteristics, COVID-19-related aspects

Table 1 depicts baseline characteristics of both groups. In comparison to the reference group, patients reported less educational years and annual household income, being single or retired more often, being less likely to work full-time, and having a smaller flat size. The number of study participants resorting to substance use as a means to feel better in the face of the pandemic increased among both groups (significant increase in the reference group only). However, at both time points of data acquisition, a significantly higher percentage of patients indicated an increased substance use.

**Table 1:** Baseline characteristics.

**Variable**	**Patients** **(*****N*** = **68)**		**Reference group** **(*****N*** = **136)**	**Statistics**	***p*** **-value**
	**Mean** ± **SD or N (%)**		**Mean** ± **SD or N (%)**		
Gender					
Male	40 (58.8%)		80 (58.8%)		1.00
Female	28 (41.2%)		56 (41.2%)		
Age (Years)	52.4 ± 11.7 (25–79)		52.2 ± 12.5 (21–96)	|Z| = 0.128	0.898
Education (Years)	12.9 ± 5.5		15.4 ± 3.7	|Z| = 4.039	<0.001
Residence					
Tyrol (Austria)	57 (83.8%)		89 (65.4%)		0.008
South Tyrol (Italy)	11 (16.2%)		47 (34.6%)		
Relationship					
Single	29 (42.6%)		25 (18.4%)		<0.001
Fixed partnership	37 (54.4%)		111 (81.6%)		
Work situation					
Full-time	19 (28.0%)		77 (56.6%)		<0.001
Part-time	6 (8.8%)		17 (12.5%)		0.491
Self-employed	4 (5.9%)		9 (6.6%)		1.00
Education/training	1 (1.5%)		2 (1.5%)		1.00
Short-time work	1 (1.5%)		0 (0.0%)		0.333
Sick leave	5 (7.4%)		0 (0.0%)		0.004
Unemployed	4 (5.9%)		1 (0.7%)		0.043
Due to COVID-19	1 (1.5%)		1 (0.7%)		1.00
Retired	22 (32.3%)		23 (16.9%)		0.019
Homemaker	3 (4.4%)		4 (2.9%)		0.688
Others	2 (2.9%)		1 (0.7%)		0.258
Household income					
<25,000 €/year	31 (45.6%)		31 (22.8%)		0.001
25,000–49,999 €/year	23 (33.8%)		55 (40.4%)		0.445
≥ 50,000 €/year	2 (2.9%)		46 (33.8%)		<0.001
Not specified	12 (17.6%)		0 (0.0%)		<0.001
Flat size (m^2^)	102.0 ± 62.6 (median 90.0)		114.9 ± 44.1 (median 110.0)	|Z| = 2.767	0.006
… per person	60.2 ± 37.9 (median 50.0)		50.2 ± 27.5 (median 45.0)	|Z| = 1.680	0.093
Garden or Balcony	61 (89.7%)		134 (98.5%)		0.007
Severe physical health problem (e.g., diabetes, cancer, etc.)	12 (17.6%)		14 (10.3%)		0.179
Use of alcohol or other substances since the outbreak of the COVID-19 pandemic in order to feel better	T1: 39.7% (27)		T1:11.0% (15)		<0.001
	T2: 48.5% (33)		T2: 26.5% (36) ↑↑		0.003
Number of patients with ICD-10 F1x.x as primary diagnosis	46 (67.6%)		–		
Number of patients with ICD-10 F1x.x as secondary diagnosis	23 (33.8%)		–		
Average years since initial diagnosis of psychiatric disorder (base 2020)	12.9 ± 13.0 (median 11.0)		–		
Average years since first in-patient treatment due to psychiatric disorder (base 2020)	9.1 ± 11.2 (median 3.0)		–		
	T1	T2			
Current treatment due to psychiatric disorder	42 (61.8%)	32 (47.1%) ↓	–		
Psychological/ psychotherapeutic treatment	28 (41.2%)	17 (25.0%) ↓	–		
Psychiatric treatment (outside a hospital)	22 (32.4%)	20 (29.4%)	–		
Psychiatric treatment (outpatient unit of a hospital)	10 (14.7%)	7 (10.3%)	–		
General practitioner	12 (17.6%)	5 (7.4%)	–		
Care facility (work)	6 (8.8%)	2 (2.9%)	–		
Care facility (living)	1 (1.5%)	2 (2.9%)	–		

↓ Significant (p < 0.05) decrease between T1 and T2 according to McNemar-test.

↑↑ Significant (p < 0.001) increase between T1 and T2 according to McNemar-test.

Within the patient group, a significant decrease in the number of subjects receiving treatment due to their psychiatric disorder was observed over time.

### Psychological distress, loneliness, and perceived social support at baseline and follow up

Table 2 depicts the findings on psychological distress, loneliness, and perceived social support. At both assessment times, significantly more patients than individuals from the reference group achieved T-scores ≥63 in six out of nine BSCL subscales (anxiety, depression, psychoticism, somatization, interpersonal sensitivity, obsessive-compulsiveness), thus suffering from clinically relevant psychological symptoms. At baseline, significantly more patients than reference participants indicated moderate loneliness, whereas at follow-up, the percentage of individuals indicating moderate loneliness had significantly *de*creased within the patient group and significantly *in*creased within the matched reference group, thus leading to a significant between-group-difference. On the other hand, the percentage of individuals indicating severe loneliness was comparable between groups at baseline and significantly higher among patients at follow-up. Regarding perceived social support from family, friends, and other significant close relationships, MSPSS mean scores were generally significantly higher in individuals from the reference group than in patients. However, the percentage of individuals highly perceiving social support was comparable between groups.

**Table 2:** Psychological distress, loneliness, and perceived social support at baseline (T1) and follow-up (T2).

**Variable**		**Patients**		**Reference group**	**Statistics**	***p*** **-value**
Psychological distress (BSCL)		*T* value ≥ 63% (*N*)	N*	*T* value ≥ 63% (*N*)		
Anger-hostility	T1 *T2*	17.6% (12) *11.8% (8)*	68	8.8% (12) *8.8% (12)*		0.063 0.450
Anxiety	T1 *T2*	35.3% (24) *26.5% (18)*	68	14.0% (19) *13.2% (18)*		<0.001 0.016
Depression	T1 *T2*	22.1% (15) *23.5% (16)*	68	7.4% (10) *6.6% (9)*		0.003 <0.001
Paranoid ideation	T1 *T2*	17.6% (12) *11.8% (8)*	68	9.6% (13) *8.8% (12)*		0.109 0.453
Phobic anxiety	T1 *T2*	41.2% (28) *27.9% (19)*	68	36.8% (50) *23.5%* (32) ↓		0.440 0.386
Psychoticism	T1 *T2*	33.8% (23) *17.6% (12)*	68	12.5% (17) *11.0% (15)*		<0.001 <0.001
Somatization	T1 *T2*	17.6% (12) *16.2% (11)*	68	7.4% (10) *5.9% (8)*		0.028 0.018
Interpersonal sensitivity	T1 *T2*	19.1% (13) *20.6% (14)*	68	6.6% (9) *5.9% (8)*		0.008 0.001
Obsessive-compulsiveness	T1 *T2*	26.5% (18) *19.1% (13)*	68	6.6% (9) *8.1% (11)*		<0.001 0.019
Global Severity Index	T1 *T2*	27.9% (19) *25.0% (17)*	68	9.6% (13) *9.6% (13)*		<0.001 0.002
Global Severity Index (Mean / ± SD)	T1 *T2*	0.66 / ± 0.54 *0.65 / ± 0.48*	62 61	0.35 / ± 0.42 *0.34 / ± 0.41*	t = −3.970; df = 1 t = −4.559; df = 1	<0.001 <0.001
**Loneliness** (TILS; range: 3–9)		% (N)		% (N)		
Moderate (TILS score 5–6)	T1 *T2*	44.1% (30) *23.5% (16)*↓	68	29.4% (40) *42.6% (58)* ↑		0.028 0.019
Severe (TILS score ≥7)	T1 *T2*	17.6% (12) *27.9% (19)*	68	15.4% (21) *11.8% (16)*		0.686 0.003
**Perceived social support** (MSPSS; range: 1–5)		**>50%** % (N) or Mean ± SD		**>50%** % (N) or Mean ± SD		
Total score	T1 *T2*	86.8% (59) *86.8% (59)*	68	95.6% (130) *94.6% (129)*		0.334 0.126
Family	T1 *T2*	3.65 ± 1.02 *3.64 ± 1.22*	67	4.26 ± 0.84 *4.26 ± 0.77*	t = 4.551; df = 1 t = 3.807; df = 1	<0.001 <0.001
Friends	T1 *T2*	3.63 ± 1.06 *3.58 ± 1.21*	65 66	4.20 ± 0.84 *4.19 ± 0.81*	t = 3.825; df = 1 t = 3.710; df = 1	<0.001 <0.001
Significant other	T1 *T2*	4.07 ± 0.94 *4.14 ± 0.99*	66 68	4.58 ± 0.57 *4.53 ± 0.59*	t = 4.094; df = 1 t = 3.032; df = 1	<0.001 0.003

*BSCL, Brief Symptom Checklist; MSPSS, Multidimensional Scale of Perceived Social Support; TILS, Three-Item Loneliness Scale*.

N* = Number of patients which the presented value is based on. For the reference group it is n = 136.

↓ Significant (p < 0.001) decrease between T1 and T2 according to McNemar-test.

↑ Significant (p < 0.001) increase between T1 and T2 according to McNemar-test.

### Associations of psychological distress with substance use, perceived social support, and loneliness

Table 3 depicts Spearman correlations between GSI scores and covariates for patients and the reference group. The perception of social support was negatively associated with psychological distress and loneliness in patients as well as the reference group, whereas a positive association was detected between psychological distress and loneliness. According to Fisher's z transformed comparison, correlation coefficients did not differ significantly between groups.

**Table 3:** Correlations between T1 and T2 variables (Spearman's rho).

**Group**	**Measure**	**GSI (T1)**	**GSI (T2)**	**TILS (T1)**
**Patients**	Psychological distress (GSI; T2)	0.727^**^		
	Loneliness (TILS; T1)	0.521^***^	0.626^***^	
	Social support (MSPSS; T1)	−0.417^***^	−0.447^***^	−0.344^**^
**Reference group**	Psychological distress (GSI; T2)	0.765^**^		
	Loneliness (TILS; T1)	0.574^***^	0.539^***^	
	Social support (MSPSS; T1)	−0.298^***^	−0.236^**^	−0.330^***^

^*^GSI, Global Severity Index; MSPSS, Multidimensional Scale of Perceived Social Support; TILS, Three-Item Loneliness Scale.

Correlation coefficients did not differ significantly between both groups according to Fisher's z transformed testing.

^*^p < 0.05;

** p < 0.01;

*** p < 0.001.

In the matched reference group, substance use as a means to feel better in the face of the pandemic was associated with higher GSI scores at T1 (Median_noconsume_ = 0.19 vs. Median_consume_ = 0.47; |z| = 3.046; *p* = 0.002) and T2 (Median_noconsume_ = 0.19 vs. Median_consume_ = 0.42; |z| = 2.570; *p* = 0.010), whereas in the patient group, this was only the case at follow-up (Median_noconsume_ = 0.49 vs. Median_consume_ = 0.72; |z| = 2.160; *p* = 0.031). In addition, higher loneliness scores were observed in individuals from the reference group using alcohol or other substances (Median_noconsume_ = 3.99 vs. Median_consume_ = 6.00; |z| = 2.054; *p* = 0.040).

Findings of repeated measures analyses of covariance are shown in Table 4. This analysis revealed significant GSI score differences between patients and the reference group (higher scores in patients) when corrected for covariates (Mean_patients_ = 0.52, S.E. = 0.049; Mean_references_ = 0.39, S.E. = 0.029). Substance use as well as loneliness were significant predictors (*p* = 0.023 and *p* < 0.001, respectively) of psychological distress. Testing for interactions between the individual predictor variables and time and/or group did not attain statistical significance.

**Table 4:** Effect of substance use, perceived social support, and loneliness on psychological distress in patient and reference group—findings of repeated measures ANCOVA (z-standardized).

		**df**	**MS**	***F***	**partial** η^2^	***p*** **-value**
**Psychological distress (GSI)**	Between-subjects effects					
	Group (patients vs. references)^†^	1	1.185	5.483	0.029	0.020
	Substance use (T1)	1	1.137	5.261	0.028	0.023
	Perceived social support (MSPSS; T1)	1	0.812	3.756	0.020	0.054
	Loneliness (TILS; T1)	1	16.290	75.337	0.292	<0.001
	Within-subjects effects					
	Time	1	0.031	0.671	0.004	0.414
	Time × Group (patients vs. references)^†^	1	0.008	0.178	0.001	0.673
	Time × substance use (T1)	1	0.065	1.415	0.008	0.236
	Time × MSPSS (T1)	1	0.129	2.799	0.015	0.096
	Time × TILS (T1)	1	0.097	2.090	0.011	0.150

† Patient group (n = 52), reference group (n = 136).

*Df, degrees of freedom; MS, mean square*.

*Interactions between group (patients vs. references) and covariates were not significant at a 5% significance level. The model including matching factors (age and sex) did not differ from the model without included matching factors*.

Findings for psychological distress at baseline and follow-up with adjustments for substance use, perceived social support, and loneliness are depicted in Supplementary Table 1. Here, significant differences were found between patients and individuals from the reference group (*GSI T1*: Mean_patients_ = 0.54, S.E. = 0.055; Mean_references_ = 0.39, S.E. = 0.033; *GSI T2*: Mean_patients_ = 0.51, S.E. = 0.053; Mean_referencess_ = 0.38, S.E. = 0.031). A feeling of loneliness at baseline was the strongest predictor of psychological distress both at baseline (β = 0.246; $\eta_{\text{p}}^{2}$ = 0.274; *p* < 0.001) and at follow-up (β = 0.211; $\eta_{\text{p}}^{2}$ = 0.232; *p* < 0.001), whereas substance use arose as significant predictor of psychological distress only at baseline, but not at follow-up. In contrast, perceived social support at baseline was a significant predictor of the GSI score at follow-up only, but not at baseline.

Regarding the results of the linear mixed model analyses accounting for COVID-19 incidence rate and time of measurement, neither of the two variables were significant explanatory factors. This held true for both Tyrol and South Tyrol. Moreover, the results of the repeated measures ANCOVA described above remained unchanged, validating the complete-case analysis (see Supplementary Table 2).

## Discussion

### General remarks

With the current report, we intended to provide an account of the repercussions the pandemic has on individuals suffering from SUD. It should be noted that baseline and follow-up assessments took place during different phases of the pandemic. The baseline survey was performed in summer and fall 2020 after the first wave of infections, at which time public health measures were relatively lax compared to the regulations in the following winter, during which follow-up took place. Thus, we consider our findings to be associated with increasing psychosocial stress surrounding the pandemic situation. However, we lack pre-COVID-19 baseline measures and accordingly, causal relationships between the pandemic and people's mental health cannot be deduced from our data.

### Substance use and its effects on wellbeing

As expected, substance use was generally significantly more prevalent among patients compared to individuals from the matched reference group. However, it rose between time points among both groups. While the increase in substance use from baseline to follow-up was significant among the reference group only, this finding supports our first hypothesis and is in line with previous research from other countries (21, 23, 26, 43). Notably, our data show that across groups, substance use significantly predicted psychological distress. This is consistent with the findings of Taylor et al. (26) who reported on an association between substance use and COVID-19-related traumatic stress symptoms. While it cannot be ruled out that both substance use and psychological distress may be associated with further factors like personality traits, resilience etc., our data suggest that an increase in substance use may constitute a maladaptive coping strategy to exceptional psychosocial stress.

### Attendance of psychiatric care among patients suffering from SUD

Our finding of reduced attendance of psychiatric and psychological/psychotherapeutic treatment facilities among SUD patients in the course of the pandemic is in line with previous findings from different countries and in varying diagnostic groups (84–88). This constitutes one of the detrimental effects of the pandemic on psychiatric care and underscores the necessity of maintaining low-threshold treatment offers.

### Psychological distress

As expected and in line with previous findings (47–51), the prevalence of clinically relevant psychological distress was significantly higher among SUD patients compared to the reference group (T1: 27.9 vs. 9.6%, T2: 25.0 vs. 9.6%). Individuals suffering from SUD generally tend to dispose of a comparatively limited amount of material and social resources (89, 90). This is reflected in the presently reported group differences in relationship status or annual household income and the more disadvantageous scores in the measures of loneliness and perceived social support. Evidence points toward a negative correlation of substance use and measures of resilience (52, 53). Thus, we expected the patient group to fare worse during the pandemic, i.e., to exhibit a comparatively larger increase in the GSI by T2. Our data did not support this hypothesis, as there was no significant effect of the time by group interaction on the GSI. As reflected in the scores of the TILS and the MSPSS, this observation might be due to the patient group enjoying less of a social network initially. Consequently, the reduction of social contacts resulting from lockdown measures could potentially not have had as profound an effect on their level of distress as among the reference group. Previous studies provide a similar picture among patients suffering from other psychiatric disorders. Pan et al. (91), for example, found that compared to healthy subjects, people with depressive, anxiety, or obsessive-compulsive disorders exhibited more pronounced psychopathology in a number of dimensions both before the pandemic and after the national lockdown in the Netherlands. Notably, symptom severity in patients increased by a lesser degree. The authors suggested greater stability encountered in everyday routines or a diminished sense of being an outsider as possible explanations, as all of society had to reduce social contacts during lockdowns (91). Similar effects could have taken place in our sample.

The prevalence of psychological distress did increase neither in SUD patients nor in individuals from the reference group over time. Previous research has shown an increase of psychological distress in the general population during the first wave of COVID-19 in spring 2020, which subsided by summer to early fall 2020 as restrictions and infection numbers were decreasing (5, 6, 44, 92). Data on psychological wellbeing during subsequent lockdowns point toward a similar mental health burden (93). Our baseline survey took place during a phase of relatively eased restrictions in 2020, while the follow-up was conducted during the second wave. Thus, a deterioration of psychological wellbeing appeared likely but could not be confirmed. This might be attributed to people having learned to adapt and cope during the first lockdown. Conversely, this observation could be connected to an ongoing sense of uncertainty and insecurity regarding people's economical and health-related outlook affecting them beyond immediate, short-term developments. Eventually, this issue cannot be sufficiently explored by the available data.

### Loneliness and perceived social support

The results of the ANCOVA provided further support for the importance of a social network for psychological wellbeing. Loneliness was a highly significant factor determining the degree of psychological distress, which is consistent with previous publications showing that increased loneliness during the pandemic was associated with an increase in psychological distress or with the intensity of psychopathology (41–45). Regarding perceived social support, our findings were less conclusive. Only in an ANCOVA considering the GSI at both time points separately, perceived social support was significantly determining the GSI at follow-up. In line with previous research (34, 35), this can be interpreted as social support being a protective factor for mental health during the pandemic.

### Limitations

A number of limitations of the present study have to be addressed. Firstly, we repeatedly observed interesting trends in our data, which failed to reach statistical significance (e.g., increased substance use and an increase in severe loneliness over time in patients). Thus, the current investigation might have profited from a larger number of participants. Also, only a fraction of the contacted patients responded and completed both surveys, not all potential study participants may have had access to the internet and thus to the online surveys, and data was entirely self-reported, which made the current investigation prone to a number of biases. In particular, the absence of previous psychiatric conditions among individuals from the reference group could not be verified independently. Moreover, we only surveyed *if* participants used alcohol or other substances in response to the pandemic, while the amount and frequency as well as the type of substances used remained unclear. Further studies are needed to investigate this issue in more detail. We defined the patient group across diagnoses with a majority having been treated for alcohol use disorder. Thus, we considered a range of different conditions as a single entity, which might have led to biased conclusions or to missing effects.

Furthermore, the current data is derived from a transnational sample. As the authorities in Austria and Italy decided independently on what measures to implement and when to do so, the psychosocial stress on participants might not have been identical in both regions. Additionally, organizational reasons caused data acquisition in South Tyrol to be delayed by several weeks and accordingly, some of the baseline data was gathered when incidence rates were on the rise. In Tyrol, relatively low case numbers had been reported during the first data acquisition period (94) and substantial tightening of measures only took place after this period had ended (95). Conversely, in South Tyrol, there was a surge in infection numbers toward the end of the respective period (96), accompanied by an escalation of public health measures including tightening of curfews, business closures and domestic travel restrictions in early November toward the end of the acquisition period. However, neither the COVID-19 seven-day incidence rate nor the time of measurement were significant explanatory factors and we therefore consider it reasonable to assume that this time lag did not have relevant effects on our findings. Still, we assume that the observed changes between time points can be sensibly attributed to pandemic-related changes. Even though the incidence rates did not prove to be an explanatory factor, public health measures and the resulting effects on social embeddedness, economic uncertainties, and personal health-related worries have changed in the course of the pandemic. They clearly constitute a psychosocial burden of multifaceted nature going beyond single descriptive parameters like the incidence rate. In contrast to the described differences during the first period of data acquisition, the pandemic situation in both subpopulations were resembling each other more closely in the second. In both regions, infection rates had reached a peak before the onset of and were consistently high throughout data acquisition with a surge of numbers in Italy in early March (94, 96). In Tyrol, a strict lockdown was in place for most of the period, while in South Tyrol, wide-ranging travel restrictions, curfews and business closures were in place throughout the respective period as well as lockdown measures over the Easter holidays in early April.

Lastly and possibly most importantly, we lacked pre-pandemic baseline data, which might have allowed more comprehensive and meaningful inferences of the pandemic's differential effect on both individuals with SUD and those without self-reported mental health disorders.

## Conclusion and outlook

Investigations of the exact ways in which substance use patterns change during crises are needed in order to gain a better understanding of how to address individuals suffering from SUD under such circumstances. The current study found both, patients and individuals from the reference group to resort to substance use as a means of coping, which underlines the need for preventive and educational measures on a societal scale. Adapting these measures to the current situation, as could be achieved by an implementation of digital strategies, is of major relevance (97, 98). Further, efforts to develop treatment schemes that allow maintaining patients under professional attendance in difficult circumstances appear crucial. As it has been indicated that online resources are an effective way to complement care in times of contact restriction (99, 100), implementing Digital Health Infrastructure appears to be a sensible approach (57, 101).

## Data availability statement

The raw data supporting the conclusions of this article will be made available by the authors, without undue reservation.

## Ethics statement

The studies involving human participants were reviewed and approved by the Ethics Commission of the Medical University Innsbruck and the Ethics Committee for Clinical Research of the Sanitary Agency of South Tyrol. The patients/participants provided their written informed consent to participate in this study.

## Author contributions

AH, BF-A, SP, BH, and BP designed the study and wrote the protocol. Recruitment was performed by FT and ACh. TS undertook statistical analysis. KL wrote the first draft of the manuscript with contributions by TS. Revision of the manuscript was carried out by TS, KL, and AH. All authors contributed to and have approved the final manuscript.

## Funding

This work was supported by the Federal State of Tyrol (Grant No. F.21427). The funder of this study had no role in study design, data collection, data analysis, data interpretation, or writing of the report.

## Conflict of interest

Author BH owns part of the IPRs of the CHES software tool. The remaining authors declare that the research was conducted in the absence of any commercial or financial relationships that could be construed as a potential conflict of interest.

## Publisher's note

All claims expressed in this article are solely those of the authors and do not necessarily represent those of their affiliated organizations, or those of the publisher, the editors and the reviewers. Any product that may be evaluated in this article, or claim that may be made by its manufacturer, is not guaranteed or endorsed by the publisher.
