# Supplementary material for: Addiction in the time of COVID-19: Longitudinal course of substance use, psychological distress, and loneliness among a transnational Tyrolean sample with substance use disorders
Source: Front Psychiatry. 2022 Jul 25;13:918465. doi: 10.3389/fpsyt.2022.918465 (PMC9380400; doi:10.3389/fpsyt.2022.918465)
Supplement: Supplementary file 1 [file Data_Sheet_1.docx]

**Supplementary Table 1: Parameter estimates of GSI at T1 and T2 with repeated measures ANCOVA (z-standardized)**

|  |  | Standardized coeff. | | t | 95% CI | | partial η^2^ | *p*-value |
| --- | --- | --- | --- | --- | --- | --- | --- | --- |
|  |  | β | S.E. |  | LB | UB |  |  |
| **Psychological Distress**  (GSI; T1) | Group (patients vs. controls)^†^ | -0.149 | 0.066 | -2.255 | -0.280 | -0.019 | .027 | .025 |
|  | Substance use (T1) | 0.077 | 0.030 | 2.528 | 0.017 | 0.137 | .034 | .012 |
|  | Perceived social support (MSPSS; T1) | -0.031 | 0.030 | -1.035 | -0.091 | 0.028 | .006 | .302 |
|  | Loneliness (TILS; T1) | 0.246 | 0.030 | 8.312 | 0.188 | 0.305 | .274 | <.001 |
| **Psychological Distress**  (GSI; T2) | Group (patients vs. controls)^†^ | -0.126 | 0.063 | -1.991 | -0.252 | -0.001 | .021 | .048 |
|  | Substance use (T1) | 0.047 | 0.029 | 1.617 | -0.010 | 0.105 | .014 | .108 |
|  | Perceived social support (MSPSS; T1) | -0.073 | 0.029 | -2.515 | -0.130 | -0.016 | .033 | .013 |
|  | Loneliness (TILS; T1) | 0.211 | 0.028 | 7.433 | 0.155 | 0.267 | .232 | <.001 |

† = Patient-Group (n = 52), Control-Group (n = 136)

Abbreviations: GSI = Global Severity Index; MSPSS = Multidimensional Scale of Perceived Social Support; TILS = Three-Item Loneliness Scale; S.E. = standard error; LB = lower bound; UB = upper bound

Note: Interactions between Group (patients vs. controls) and covariates were not significant at a 5% significance level

***Supplementary Table 2: Estimates of fixed effects in the Linear Mixed Model with AR(1) covariance structure including GSI as dependent variable and z-standardized covariates***

|  | | Standardized coeff. | | t | 95% CI | | *p* |
| --- | --- | --- | --- | --- | --- | --- | --- |
|  |  | β | S.E. |  | LB | UB |  |
| **Factors** | Patients vs. controls (Group) | 0.221 | 0.060 | 3.660 | 0.102 | 0.340 | <.001 |
|  |  |  |  |  |  |  |  |
|  | MoP – July 2020 | -1.785 | 1.839 | -0.971 | -5.403 | 1.833 | .332 |
|  | MoP – August 2020 | -1.119 | 1.811 | -0.618 | -4.683 | 2.445 | .537 |
|  | MoP – September 2020 | -0.676 | 1.548 | -0.437 | -3.721 | 2.369 | .663 |
|  | MoP – October 2020 | -1.497 | 1.469 | -1.020 | -4.387 | 1.392 | .309 |
|  | MoP – November 2020 | -2.770 | 2.356 | -1.176 | -7.410 | 1.870 | .241 |
|  | MoP – December 2020 | -1.560 | 1.517 | -1.029 | -4.544 | 1.424 | .304 |
|  | MoP – January 2021 | -1.123 | 1.552 | -0.724 | -4.176 | 1.930 | .470 |
|  | MoP – February 2021 | -1.412 | 1.469 | -0.961 | -4.302 | 1.478 | .337 |
|  | MoP – March 2021 | -1.100 | 1.351 | -0.814 | -3.758 | 1.559 | .416 |
|  | MoP – April 2021 (reference) | 0^a^ | 0^a^ | - | - | - | - |
|  | |  |  |  |  |  |  |
| **Covariates** | Substance use | 0.116 | 0.043 | 2.679 | 0.031 | 0.201 | .008 |
|  | Perceived social support (MSPSS) | -0.080 | 0.023 | -3.525 | -0.125 | -0.036 | <.001 |
|  | Loneliness (TILS) | 0.158 | 0.020 | 8.051 | 0.119 | 0.196 | <.001 |
|  | Residence (Austria vs. Italy) | -0.526 | 0.567 | -0.928 | -1.642 | 0.590 | .354 |
|  | COVID-19 IR | 6.347 | 7.732 | 0.821 | -8.861 | 21.555 | .412 |
| **Interactions** |  |  |  |  |  |  |  |
|  | MoP (July 2020) × Residence | 0.568 | 0.593 | 0.958 | -0.599 | 1.735 | .339 |
|  | MoP (August 2020) × Residence | -^b^ | -^b^ | - | - | - | - |
|  | MoP (September 2020) × Residence | 0.305 | 0.587 | 0.520 | -0.851 | 1.462 | .604 |
|  | MoP (October 2020) × Residence | 0.536 | 0.568 | 0.945 | -0.582 | 1.655 | .346 |
|  | MoP (November 2020) × Residence | -^b^ | -^b^ | - | - | - | - |
|  | MoP (December 2020) × Residence | 0.579 | 0.599 | 0.968 | -0.600 | 1.759 | .334 |
|  | MoP (January 2021) × Residence | 0.295 | 0.622 | 0.474 | -0.931 | 1.520 | .636 |
|  | MoP (February 2021) × Residence | 0.505 | 0.579 | 0.872 | -0.636 | 1.645 | .384 |
|  | MoP (March 2021) × Residence | -^b^ | -^b^ | - | - | - | - |
|  | MoP (April 2021) × Residence (reference) | 0^a^ | 0^a^ | - | - | - | - |
|  |  |  |  |  |  |  |  |
|  | MoP (July 2020) × COVID-19 IR | -6.580 | 7.842 | -0.839 | -22.003 | 8.844 | .402 |
|  | MoP (August 2020) × COVID-19 IR | -7.417 | 7.822 | -0.948 | -22.803 | 7.969 | .344 |
|  | MoP (September 2020) × COVID-19 IR | -5.641 | 7.802 | -0.723 | -20.986 | 9.704 | .470 |
|  | MoP (October 2020) × COVID-19 IR | -6.225 | 7.731 | -0.805 | -21.432 | 8.982 | .421 |
|  | MoP (November 2020) × COVID-19 IR | -4.231 | 7.913 | -0.535 | -19.794 | 11.333 | .593 |
|  | MoP (December 2020) × COVID-19 IR | -6.450 | 7.732 | -0.834 | -21.659 | 8.758 | .405 |
|  | MoP (January 2021) × COVID-19 IR | -6.516 | 7.734 | -0.842 | -21.728 | 8.697 | .400 |
|  | MoP (February 2021) × COVID-19 IR | -6.382 | 7.732 | -0.825 | -21.591 | 8.826 | .410 |
|  | MoP (March 2021) × COVID-19 IR | -7.859 | 7.948 | -0.989 | -23.492 | 7.775 | .323 |
|  | MoP (April 2021) × COVID-19 IR (reference) | 0^a^ | 0^a^ | - | - | - | - |

Abbreviations: MSPSS=Multidimensional Scale of Perceived Social Support; TILS=Three-Item Loneliness Scale; MoP=Month of Participation; IR=7-day Incidence Rate; S.E.=standard error; LB=lower bound; UB=upper bound

^a^ This parameter is set to zero because it is redundant.

^b^ The interaction cannot be calculated due to missing information about “Residence”
